# Designing Health Websites Based on Users’ Web-Based Information-Seeking Behaviors: A Mixed-Method Observational Study

**DOI:** 10.2196/jmir.5661

**Published:** 2016-06-06

**Authors:** Patrick Cheong-Iao Pang, Shanton Chang, Karin Verspoor, Jon Pearce

**Affiliations:** ^1^ Department of Computing and Information Systems The University of Melbourne Parkville Australia; ^2^ NICTA Victoria Research Lab Melbourne Australia; ^3^ Health and Biomedical Informatics Centre The University of Melbourne Melbourne Australia

**Keywords:** consumer health information, public health informatics, exploratory behavior, hypermedia

## Abstract

**Background:**

Laypeople increasingly use the Internet as a source of health information, but finding and discovering the right information remains problematic. These issues are partially due to the mismatch between the design of consumer health websites and the needs of health information seekers, particularly the lack of support for “exploring” health information.

**Objective:**

The aim of this research was to create a design for consumer health websites by supporting different health information–seeking behaviors. We created a website called *Better Health Explorer* with the new design. Through the evaluation of this new design, we derive design implications for future implementations.

**Methods:**

Better Health Explorer was designed using a user-centered approach. The design was implemented and assessed through a laboratory-based observational study. Participants tried to use Better Health Explorer and another live health website. Both websites contained the same content. A mixed-method approach was adopted to analyze multiple types of data collected in the experiment, including screen recordings, activity logs, Web browsing histories, and audiotaped interviews.

**Results:**

Overall, 31 participants took part in the observational study. Our new design showed a positive result for improving the experience of health information seeking, by providing a wide range of information and an engaging environment. The results showed better knowledge acquisition, a higher number of page reads, and more query reformulations in both focused and exploratory search tasks. In addition, participants spent more time to discover health information with our design in exploratory search tasks, indicating higher engagement with the website. Finally, we identify 4 design considerations for designing consumer health websites and health information–seeking apps: (1) providing a dynamic information scope; (2) supporting serendipity; (3) considering trust implications; and (4) enhancing interactivity.

**Conclusions:**

Better Health Explorer provides strong support for the heterogeneous and shifting behaviors of health information seekers and eases the health information–seeking process. Our findings show the importance of understanding different health information–seeking behaviors and highlight the implications for designers of consumer health websites and health information–seeking apps.

## Introduction

The Internet has been widely used for accessing health information [1]. However, finding and discovering the right and useful information remains problematic [2-5]. Despite the efforts made to improve the provision of online health information, some complications still exist for the health information seeker, such as, inefficiently using search engines [6-9], lacking the cognitive skills and health literacy for searching [10-13], and feeling disengaged with health websites due to usability and design issues [14,15]. These problems add burdens to health information seekers and reduce the overall user experience.

As discussed in prior work, one of the reasons behind these problems is that the design of health websites does not address the needs of health information seekers [16-19]. Depending on the specific scenario, seekers may demonstrate either focused or exploratory search approach in the information seeking process [17,20,21]. With different search approaches, seekers use different strategies to find information, which need to be supported with features such as tools for exploratory search, reading-friendly user interface, and memory aids for reviewed information [19]. These features or considerations are often missing in the design of health websites and health information–seeking apps.

To understand and support health information–seeking behaviors, a website called Better Health Explorer (BHX) was designed using a user-centered design approach. The design is based on the conceptualization of search approaches and a classification of health information–seeking behaviors [19]. In this paper, we aim to investigate what improvements can be made to the current tools for health information seeking, by using BHX as the vehicle of a human-based evaluation.

This project adopted a mixed-method approach. Overall, 31 participants took part in an observational study of using BHX and an existing live health website. Overall, our proposed design shows a positive result in improving the user experience of health information seeking. The results show better knowledge acquisition, a higher number of page reads, and more query reformulations in different search tasks. Moreover, participants spend more time to discover health information in exploratory search tasks with our design. In addition, we summarize 4 design considerations for designing consumer health websites and health information–seeking apps, namely: (1) providing a dynamic information scope; (2) supporting serendipity; (3) considering trust implications; and (4) enhancing interactivity.

We demonstrate that BHX improves the support for heterogeneous and shifting behaviors of health information seekers and eases the health information–seeking process. We therefore recommend that designers, Human-computer interaction (HCI) practitioners, and researchers consider these findings in the design of consumer health websites and health information–seeking apps.

## Methods

This section will discuss the different aspects of our study. First, we will introduce the rationale and the implementation of BHX, which was the website used in our study. Then, we will explain the design of our user study and finally, the methodology for data analysis.

### Better Health Explorer

Better Health Explorer is not only a new user interface (UI) for health information seeking but also a vehicle for evaluating the design and highlighting the considerations for designing consumer health websites. In this subsection, we will start with a brief introduction of the theoretical framework that underpins the design, followed by a short description of UI features that reflect this framework in the implementation of BHX.

#### Theoretical Background

The design process of BHX follows a user-centered approach. Research has proposed to use user-centered design for eHealth technologies and informational websites [22,23]. In our prior work [17-19], we have conceptualized both the search approaches used in health information seeking and the health information–seeking behaviors demonstrated by different seekers. We have learnt that seekers adopt differing search approaches for different scenarios, and thus, the selected search approach affects the actual health information–seeking behavior. This theoretical work underpins the design of BHX. The following paragraphs will give a brief explanation about this conceptualization.

##### Focused and Exploratory Search

**Figure 1:**
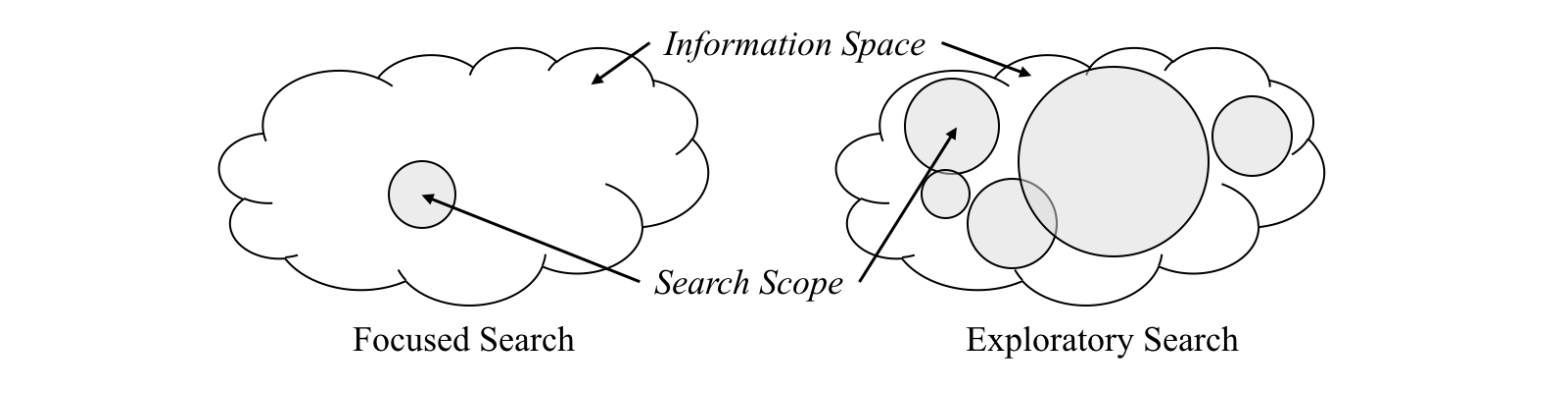
An illustration of focused and exploratory search approaches.

We have identified 2 search approaches in the process of health information seeking, namely focused search and exploratory search [24,25]. In the health context, we have found that people demonstrate both types of search approaches depending on a number of factors, for instance, their level of knowledge about the health problem, their levels of curiosity, the perceived situational relevance to the health problem, and so forth [17]. Figure 1 illustrates the 2 search approaches and their differences.

Searching with precise keywords and iteratively narrowing down the search scope are sample activities of focused search. As shown in Figure 1, focused seekers concentrate on a small range of information. They often have better knowledge about the health problem and a clearer idea of what they are looking for [17]. However, this approach is difficult for many seekers, as not everyone can clearly express the health issue through search queries or use accurate terminology [7,8].

Exploratory search is another search approach found in health information seeking. Exploratory seekers often are unfamiliar with the knowledge domain and feel unsure about the search goal [24,26]. In these cases, exploratory search often arises, along with an unclear search target, as well as a wider and sparse search scope [17,19], as illustrated in Figure 1. Exploratory search also introduces learning and investigative activities beyond simply finding particular information [25], to clarify the problem and gain an overview of the situation [17].

##### Health Information–Seeking Behavior

**Figure 2:**
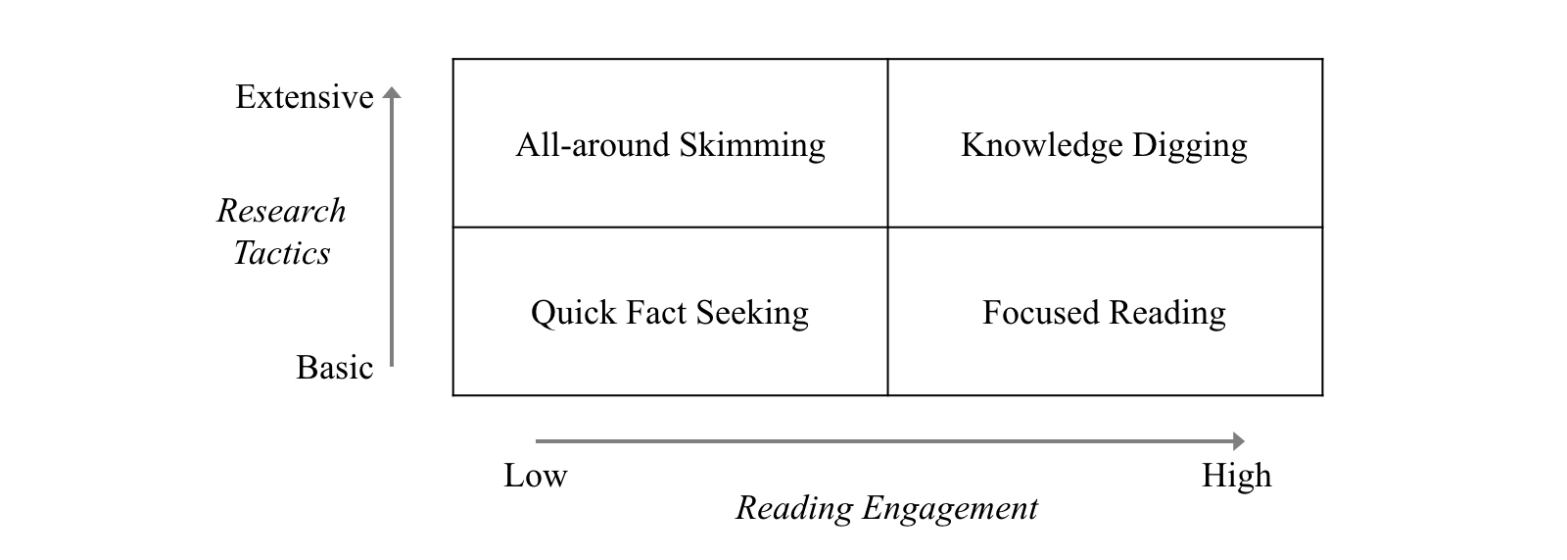
The classification of health information–seeking behaviors.

Motivated by diverse scenarios and different search approaches, health information seekers implicitly expose 4 different behaviors. As seen in Figure 2, health information–seeking behaviors can be represented with 2 dimensions: Reading Engagement and Research Tactics [19]. Reading Engagement states that a seeker prefers to commit either long or short time for reading, whereas Research Tactics captures that a seeker intends to gain a comprehensive understanding or merely seeks basic facts about the health problem.

This classification is not used for dividing individual health information seekers into different groups but for understanding their behaviors as a whole. Therefore, Figure 2 shows the range of potential behaviors users might engage in when seeking online health information. This classification can assist designers in making apps that are sensitive to the variety of user behaviors as it provides a lens for understanding all the possible behaviors that can be observed within a health website. In addition, this information is important for creating UI elements for different types of searches and providing suitable information for the diverse behaviors. Nevertheless, the motivations and the context of individual behavior are not the focus of this research.

From the design perspective, each of these behaviors leads to different requirements for user interactions with health websites. For example:

- *Quick Fact Seeking* refers to retrieving the superficial information for a specific health topic and terminating the search once it is found. For this type of behavior, websites should provide key points and a brief summary relevant to the topic.
- *All-Around Skimming* goes through a wide range of information in a fast manner. Excerpts and previews will be helpful to support this behavior, for determining what content is useful within a potentially large number of search results.
- *Focused Reading* denotes concentrated reading on a particular topic. As lengthy reading is involved in this case, reader-friendly features (eg, larger font size, bookmarking, highlighting, and so forth.) are recommended to support this behavior.
- *Knowledge Digging* indicates the intense reading associated with the in-depth research on a number of diverse health topics. Providing a broader range of information can assist users to investigate from multiple perspectives.

With this model, UIs can be designed and built by understanding the user actions associated with these behaviors [18,27].

##### Changeable Search Approaches and Behaviors

Health information seekers do not adhere to a single search approach or behavior in their search processes. Instead, they choose the approach that is most appropriate to them, based on the circumstances, the context, the urgency of the health issue, the situational relevance, and personal preferences [17,19,28]. Therefore, it is not feasible to design for just a single or a limited number of seeker types, but rather, we should focus on the properties of each health information–seeking behavior, and support such properties through the design. In this way, the design will cover most of the actions executed by each type of health information–seeking behavior.

In this subsection, we have introduced different health information–seeking behaviors and their implications for website design. The next subsection will present the actual website implementation, guided by this theoretical framework.

#### Design Considerations

Rather than replicating the work of sophisticated keyword-based search engines, the design goals of BHX are to address the needs of health information seekers and to deliver an interactive and engaging experience in the health information–seeking process. We will introduce briefly about this website in the following section. Readers can obtain the details of BHX features in another paper [18]. A video demonstration of BHX can also be found in (Multimedia Appendix 1).

**Figure 3:**
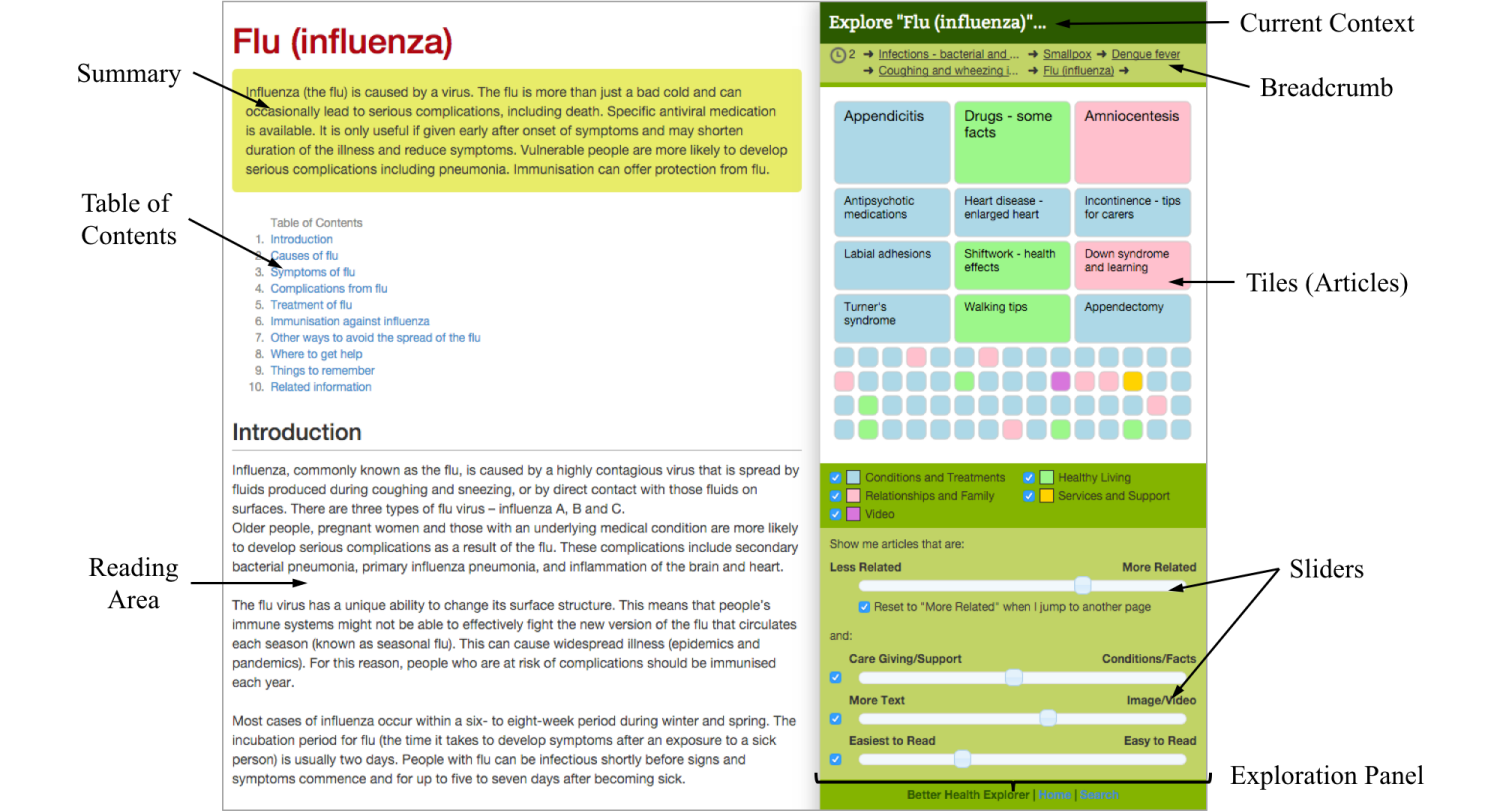
The user interface of Better Health Explorer.

Figure 3 displays a screenshot of BHX. The UI looks similar to an ordinary health website, but the exploration panel on the right gives a different experience of finding and exploring health information. Information exploration is facilitated by the list of *tiles* (top right of the screen) and *sliders* (bottom right of the screen).

Sliders are used for generating and refining queries. Health information articles that match the criteria are displayed as colored tiles at the top right of the screen. Colors denote the category that the information belongs to. Therefore, the color pattern offers an overview of the composition of the results. The keyword-less approach brought by the sliders can reduce the cognitive load of looking for health information.

In prior studies, we have learnt that health information seekers use mainly 4 different criteria to seek information [17,19]. Four sliders corresponding to these criteria are provided. In this way, the website can provide a broad range of information with hundreds of combinations of slider values based on the context currently being viewed. This satisfies the needs of different search approaches and behaviors. Meanwhile, the elimination of keyword search resolves the difficulties of generating new search queries.

Common UI elements can assist in health information seeking. The summary and the table of contents can provide an overview and structure of an article to seekers. These features are useful for behaviors of low Reading Engagement. Besides, the “breadcrumb” history bar can reflect the initial goal and the path of the search session. This allows backtracking quickly, understanding, and adjusting the goal in the progress of the information exploration [24].

An example of a concerned mother can further explain the innovative experience of seeking health information. The mother wants to find some information about a common cold for her child, and she looks for it in BHX. After some initial reading about colds, she desires to learn more around this topic and therefore adjusts the sliders. As the sliders change, the tiles start to move and jostle their positions. Her attention is grabbed by an item labeled “pneumonia”. She clicks on it, and the reading area is updated with information about pneumonia. Meanwhile, a new set of tiles pops up based on the new topic (pneumonia), so that she can explore further. The “journey” of exploration repeats again with similar steps.

#### Content

The content used in BHX is obtained from Better Health Channel (BHC) [29], which is a consumer health website established by the Victorian State Government in Australia. The dataset covers more than 250 health and medical topics for the general public. In addition to text-based materials, pictures and figures from the BHC site, as well as video clips published in their YouTube channel, are included in this study.

### Study Design

The study aims to understand how the design of BHX supports health information seeking and information exploration within consumer health websites and to summarize design considerations for future websites. We used a mixed method approach for this study, capturing both qualitative and quantitative data. This research was approved by the university’s human ethics committee.

**Figure 4:**
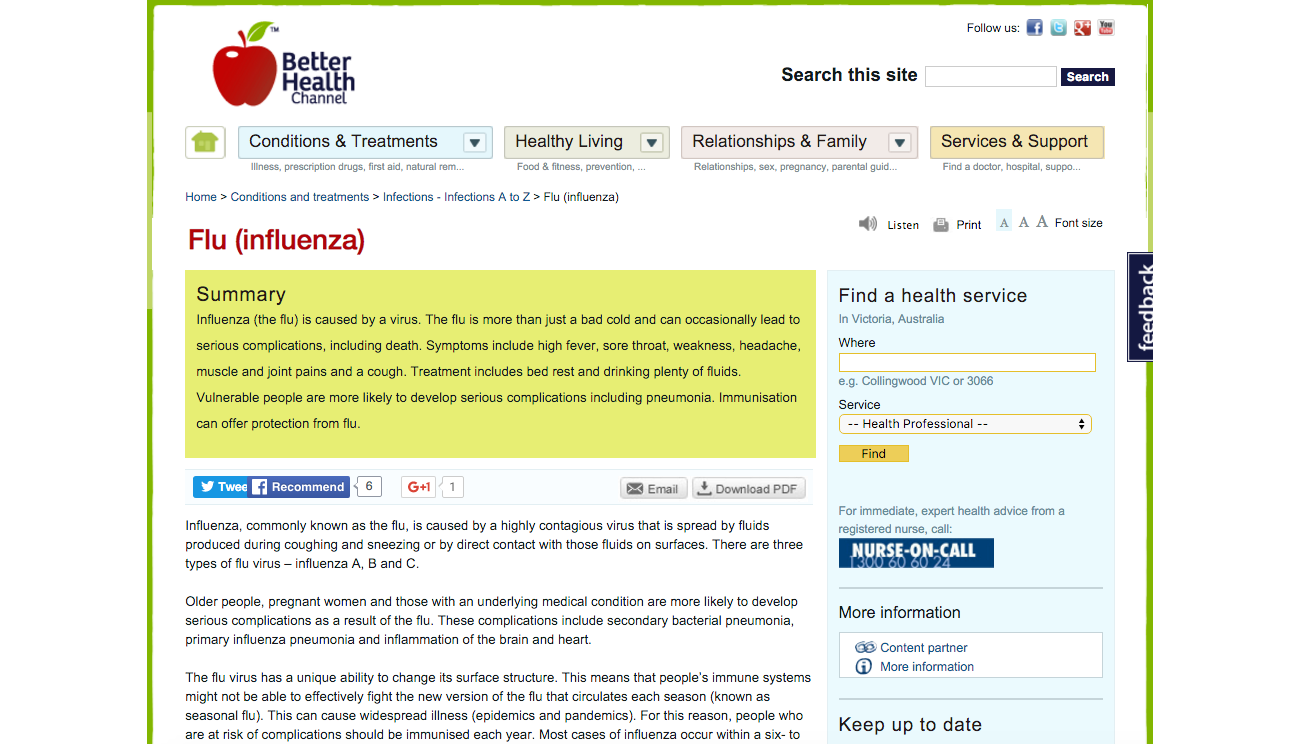
The appearance of the baseline website used in this study.

Volunteers were invited to use BHX in a laboratory-based observational study from September to October 2015. For the sake of comparison, they also used BHC, which is an existing live website and also the source of health-related content in this study. Figure 4 shows the UI of BHC. Data were recorded from the participants' use of each website, BHX and BHC, for the assigned study tasks, to assess the relative impact of the BHX design. The design of this study is similar to that of a number of other studies on health information seeking, which conducted observational studies [11,15,30-32].

#### Search Tasks

Participants were given 4 tasks consisting of 2 focused and 2 exploratory search tasks (Table 1), to observe the differences between the 2 search approaches. The tasks posed fictional health scenarios and questions about the scenarios, and we asked the participants to find answers for these questions from either website. We varied the health conditions in the task descriptions that were assigned to individual participants for their search tasks (variables underlined in Task A and B), avoiding conditions that participants may have prior knowledge of, based on their self-reported information (Multimedia Appendix 2). This particular setup was to avoid repeats when testing different websites and to minimize the potential impact of prior knowledge affecting the outcomes, for example, if the participants had suffered from that particular sickness before.

**Table 1:** Task descriptions used in the study.

Task	Website used	Type^a^	Description
A	Better Health Channel (Baseline)	F	Imagine one of your family members has recently been diagnosed with Type 2 Diabetes/hypertension. As you’re living together, your daily life might need to be changed in different ways as well. Please identify three kinds of changes that might be needed in your everyday life.
B	Better Health Explorer	F	Imagine one of your close friends has recently been diagnosed osteoporosis/asthma. As you’re living together, your daily life might need to be changed in different ways as well. Please identify three kinds of changes that might be needed in your everyday life.
C	Better Health Channel (Baseline)	E	Imagine you are going to a party and will discuss health information with your friends. Use the website provided by us to identify some interesting health topics. Continue reading until you think it is enough for the discussion.
D	Better Health Explorer	E	Imagine you are going to a party and will discuss health information with your friends. Use the website provided by us to identify some interesting health topics. Continue reading until you think it is enough for the discussion. (Same as Task C)

^a^E: exploratory search task; F: focused search task.

The design of these tasks was purposefully considered. The health issues used in the task descriptions were sourced from the most popular searched keywords from the BHC website. This ensured that the health issues were common and realistic, and the data source contained a substantial amount of information for searching. Also, the contrastive setup with 2 search approaches could facilitate the observation of their differences [33].

The design of exploratory tasks followed the principles outlined in a study by Wildemuth and Freund [34]. The scenario involving social discussions was also found to be helpful for generating exploratory search [35]. In addition, the motivation of searching for family members (Task A) and close friends (Task B) is similar as the perceived situational relevance to the seeker is high in both the cases [17].

#### Procedure

Participants began with a brief introduction to the study and the 2 websites used, followed by a demographic questionnaire. Informed consent was obtained through a signed consent form. At this stage, participants had to select one of the tasks that they had no prior experience with. Search tasks were then conducted on a desktop computer.

Participants sequentially carried out the search tasks on a desktop computer in a defined order. For counterbalancing learning and ordering effects, the order of the tasks was allocated by a 4×4 Latin Square [36]. Each task started with the home page of the website (depending on the website corresponding to the task), which consists of a list of popular health topics and a search input box. Participants were allowed to search, navigate, and browse the website freely. The only restriction was that they could not open and use websites other than the testing one. Screen captures, activity logs, and Web browsing histories were recorded for analysis.

At the halfway point and at the completion of the study session, a short semistructured interview was conducted, mainly for collecting verbal feedback about the tasks. This also gave the researcher a chance to collect feedback from the users about their experiences. The interview questions can be seen in Multimedia Appendix 3. Interviews were recorded and transcribed for future analysis.

#### Participant Recruitment

Participants were recruited via multiple channels, such as a University of Melbourne mailing list, electronic bulletin boards, and fliers posted in student lounges. We also used social media such as Facebook and Twitter to increase the exposure. We sought participants who were over aged 18 years and possessed previous experience of searching information on the Internet for comparing the 2 websites. Participants received no incentive to take part in the research.

### Data Analysis

Multiple methodologies were used to analyze the various data obtained in this study. For qualitative data, semistructured interviews were transcribed and processed with content analysis [37,38]. Themes obtained from the analysis were used to investigate the influence on the design. In addition, screen recordings were reviewed and coded by the researcher [39,40]. This information was used to categorize and compare the patterns of health information–seeking behaviors in both the websites.

For quantitative analysis, we selected 4 metrics from previous research for measuring user interactions in the health information–seeking process [24,41,42]. Page reads and task duration reflect the amount of information accessed and the engagement with the website. Clicking on links (and tiles) indicates the effort of in-depth understanding about a topic and represents the depth of search. Query reformulation is an essential concept in exploratory search for measuring the degree of information exploration [43,44]. In the context of this study, query reformulation refers to issuing a new search query in the baseline website or adjusting the sliders in BHX.

Statistical calculations on the quantitative data were performed using R version 3.2.3. We applied Wilcoxon signed-rank test [45] to verify the statistical significance and compute the effect size between the baseline and BHX in each category (ie, focused and exploratory) of search tasks. This test does not require the normality of data [46].

## Results

### Participants

Overall, 31 participants took part in the study (N=31). Among the participants, 15 (48%) were male, and 16 (52%) were female. The average age was 33.9 (standard deviation=12.67, median=29) with the range from 20 to 72. Regarding the source of recruitment, 19 (61%) reported being students in the university; 10 (32%) were staff of the university; and 2 (7%) were recruited externally.

### Health Information–Seeking Behaviors

Table 2 summarizes the annotations of health information–seeking behaviors (Figure 2) from the review of screen captures. For focused search tasks (Tasks A and B), most seeking behavior was consistent with Quick Fact Seeking (42% and 52%, respectively). However, more than half of participants demonstrated All-around Skimming (58%) in both the exploratory tasks (Tasks C and D). The results reinforce our previous results [19,27] that seekers adopt different information–seeking behaviors due to the different nature of search tasks.

**Table 2:** Seeking behaviors observed in our participants.

Seeking behavior	Focused search, n (%)		Exploratory search, n (%)	
	Task A (Baseline)	Task B (BHX^a^)	Task C (Baseline)	Task D (BHX)
Quick Fact Seeking	13 (42)	16 (52)	4 (13)	3 (10)
Focused Reading	8 (26)	5 (16)	2 (6)	2 (6)
All-around Skimming	9 (29)	9 (29)	18 (58)	18 (58)
Knowledge Digging	1 (3)	1 (3)	7 (23)	8 (26)
Total	31 (100)	31 (100)	31 (100)	31 (100)

^a^ BHX: Better Health Explorer

The figures display a distinct pattern in terms of the dimension of Research Tactics in our model of health information–seeking behaviors (Figure 2). Seekers demonstrated different behaviors across the task type, even with the same website. In focused search tasks, most participants (68% in both Tasks A and B) adopted basic Research Tactics (ie, Quick Fact Seeking and Focused Reading) behaviors. However, large portions of participants (81% in Task C; 84% in Task D) showed extensive Research Tactics (ie, All-around Skimming and Knowledge Digging) in exploratory search tasks. Nevertheless, all 4 types of information-seeking behaviors were observed in the study. This reflects the diverse composition of health information seekers in the population of health website users.

### User Interactions

Participants illustrated different levels of user interactions across focused and exploratory tasks. In focused search tasks (Tasks A and B), we did not observe significant differences between the baseline and BHX (Table 3). Participants read a similar number of pages, clicked on a similar number of links, and spent a similar amount of time in both the websites. Nevertheless, the number of query reformulations is substantially higher for BHX with a mean of 3.3 compared with 0.8 for the baseline (*P*<.001).

**Table 3:** User interaction figures in focused search tasks.

	Task A (Baseline)	Task B (BHX^a^)	Wilcoxon signed-rank test		
	Mean (SD)	Mean (SD)	Z	*P*	r
Page reads	2.7 (1.5)	2.7 (1.9)	0.230	.847	.029
Task duration (seconds)	285 (115)	271 (142)	−0.598	.558	−.076
Clicks on links (baseline)/tiles (BHX)	2.6 (1.7)	2.5 (2.0)	−0.109	.927	−.014
Query reformulation	0.8 (0.8)	3.3 (3.5)	3.942	.000^b^	.501
					

^a^ BHX: Better Health Explorer

^b^*P*<.001

However, the figures demonstrate a different pattern in exploratory search tasks (Table 4). BHX users presented a substantial higher number of pages read (*P*<.001) and more query reformulations with a large effect size (*P*<.001). They also spent more time on exploring information (*P*=.034), and followed up more links in the website (*P*<.001).

**Table 4:** User interaction figures in exploratory search tasks.

	Task C (Baseline)	Task D (BHX^a^)	Wilcoxon signed-rank test		
	Mean (SD)	Mean (SD)	Z	*P*	r
Page reads	3.9 (1.7)	5.7 (2.5)	3.986	.000^b^	.506
Task duration (seconds)	364 (153)	410 (154)	2.107	.034^c^	.268
Clicks on links (baseline) or clicks on tiles (BHX)	2.7 (1.9)	4.8 (2.4)	3.857	.000^b^	.490
Query reformulation	0.3 (0.6)	4.2 (3.3)	4.584	.000^b^	.582

^a^BHX: Better Health Explorer

^b^*P*<.001.

^c^*P*<.05.

The raw results of the quantitative figures can be obtained from Multimedia Appendix 4.

### Qualitative Feedback

This subsection presents the qualitative feedback from the participants, interpreted together with the *in situ* observations of the primary researcher. After qualitative content analysis, we have derived 4 main themes for our design.

#### Dynamic Information Scope

Participants reported that our design helped them to seek information within a dynamic information scope. Most of the time they looked for the directly relevant information, but, in certain cases, they also needed to broaden to other topics. Participants reported that BHX was useful in such cases to discover topics. This was accomplished by adjusting the sliders to allow diverse topics to appear. In contrast, traditional health websites and search engines require issuing new search queries to achieve the same result. Our approach lowered the cognitive load of constructing new search queries and thus was perceived easier to use.

It (BHX) did help me find topics, and I think here I found topics that were related to what I was reading… In another one (the baseline website) I need to look for topics. This one (BHX) showed me topics. So it is easier.

Participant #26

People like to see the context, (and) this one (BHX) shows me the context.

Participant #8

This (BHX) is very good for discovering related information.

Participant #14

I expect things that are somehow relevant.

Participant #6

Moreover, participants appreciated that BHX showed diverse types of information around the topic, which provided opportunities for approaching the problem from different perspectives.

It (BHX) gives me more options about the topic.

Participant #15

By giving you choices rather than just coming up with the top things, I think it makes (the system) more interesting to use.

Participant #3

There are a range of things that are sort of related… and things are not much related. There are a range of different aspects. So I think that has a good diversity.

Participant #3

#### Serendipity and Curiosity

Serendipity, meaning that people are surprised by seeing valuable things that they have not thought of [47], was found critical in this study. Serendipitous findings happened when seekers did not know much about the health topics, and in such a case, BHX reminded them the existing information they did not consider.

Giving me different options that I did not consider.

Participant # 15

It is useful if I can get other information connecting (a sickness) to other subjects that I have not thought of before.

Participant # 23

I never thought about this and then (it showed up). Ah! This is good.

Participant # 26

Curiosity was also an important factor for engaging users in the information exploration process. The design of BHX was observed to stimulate curiosity in the health information–seeking process.

It (BHX) is engaging because you can kinda play with it and see what you get. You have a reasonable expectation what sort of things you are going to get and what exactly you are getting out of it. It is sort of curious.

Participant # 13

I am curious to see what it is all about.

Participant # 18

Because when you started reading something, the another one (tile) gave me more options—something that captured my eyes and it is interesting.

Participant # 3

#### Trust Issues

BHX offers a “fuzzy” approach for seeking health information that is very different from search engines, which always provide best matches to the search terms. In this study, we observed that participants had different opinions about the sometimes unexpected results displayed. The following section includes some positive feedback about this approach.

(When seeing something unexpected came up…) I trust your system! I do not necessarily think that means something is wrong. I think that means maybe there is a connection there (which) I was not aware of. It is more interesting rather than a problem.

Participant # 20

This (an unexpected thing) is the information that I do not have in my mind, so it was put there like opportunities of knowledge. Because I was not aware, it gives me awareness of things that are related to the topic. Many times I do not know what to look for. I think it gives me awareness.

Participant # 15

Other participants suggested that the unexpected results would be a problem and these endangered the trust between the user and the website. The primary goal of any design should offer the best results as possible.

That makes me worry. You start to bring up information which is perhaps gonna scaring people as well. It is about to get the right stuffs to the top first.

Participant # 10

(The accuracy) It is difficult to judge for me. Unless... for example, if I am an expert of a particular topic, then I can actually make a correct call, whether this is giving me the correct information.

Participant # 23

#### Delightful User Experience

We noticed that the design of BHX delivered a delightful experience to the users. The interactivity in the information exploration process gave them the feeling of gaining more control over the website and thus increased their satisfaction. Also, the opportunities for acquiring new knowledge led to a higher engagement between the participants and the website.

The interactive nature of it… You have more control over whatever information you get.

Participant #24

Exploring using this website (BHX) is easier. You have more options. Whereas the other website (the baseline website) is fixed, (where) you do not have much to control.

Participant #5

It is good because it makes me feel more knowledge after clicking here and finding something.

Participant #15

I do enjoy using this (BHX). Because there are some interesting topics here. I am definitely getting interesting topics here. There are many many topics popping up which I am happy to follow up. There are so many I can choose from.

Participant #6

## Discussion

### Principal Findings

In this study, we have identified the combinations of heterogeneous behaviors in the health information–seeking process, in terms of different behaviors and user interactions. Overall, our design improves the user experience of health information seeking and better supports the needs of health information seekers.

The results provide insights about our innovative design for health information seeking. Participants enjoyed using the interactive UI to find information. Such a design was considered to provide a more diverse and dynamic information scope. Meanwhile, the evaluation illustrates that a design that encourages serendipitous findings can provide hints for further search directions, when people have little prior knowledge about the health topic. Interactivity and having control over the system are also factors that engage with information seekers. In terms of quantitative analysis, BHX was shown to perform better in exploratory tasks across all metrics. This highlights the importance of directly supporting information exploration in health information seeking.

These findings imply that the design of BHX is superior in supporting the various health information–seeking behaviors. In the data analysis process, we have identified 4 design considerations that are crucial for health websites and health information–seeking apps, including: (1) providing a dynamic information scope; (2) supporting serendipity; (3) considering trust implications; and (4) enhancing interactivity. We argue that these aspects play a role in the design of consumer health websites and health information–seeking apps, for providing a better environment for seeking and conveying health information.

In the following subsections, we will further discuss these design considerations with the lessons learnt from this experiment.

#### Providing a Dynamic Information Scope

For both focused and exploratory search tasks, we have identified that seekers demand information from a dynamic information scope. Although seekers often prefer to retrieve direct and relevant information to the current context, they would like to see more diverse options (eg, information of other topics but still relevant) at the same time. This helps seekers to understand the health issues from different perspectives.

We propose that the variance is an outcome of the coexistence of both focused and exploratory search approaches in health information seeking [17,19] and related to the uncertainty that arises from the health issue [17,24,48]. When possessing only little knowledge about the health problem, seekers will have an unclear search target. As a result, they look for more different options for learning and setting up a clear direction to handle the scenario. After reading enough information, the search scope turns into a more focused one because the uncertainty is reduced. The same level of diversity is not required at the point when seekers have low uncertainty.

In addition, the nature of search tasks affects information needs and the search approach used. An ill-formed and widely open health scenario, such as a self-initiated search about certain symptoms, leads to a more exploratory search and thus requires a wider range of health information. However, a search after a diagnosis often leads to a focused search because of a clear, predefined search goal.

BHX uses a combination of sliders to facilitate exploration in a dynamic information scope. Both themes from the qualitative analysis (Dynamic Information Scope and Delightful User Experience) and quantitative results reflect the success of this design component. Although many traditional health websites provide search functions, few allow exploratory search through offering a broad and dynamic range of information. To explore a new topic, users have to issue new search queries. In contrast, seekers using BHX can adjust the sliders and review the results that emerge with the new criteria. This process is more convenient, and the cognitive workload is lower compared with that while making a new search query.

Based on the aforementioned observations, we consider that access to a dynamic information scope is critical for health information seeking, as health information seekers demonstrate complicated needs and heterogeneous behaviors throughout their searches.

#### Supporting Serendipity

Serendipity, which refers to scenarios of discovering useful information with randomness and pleasure [49], can be a factor to assist in the information seeking process [25,50]. Some existing work has pointed out that health information seeking involves certain degree of serendipity [28,51], resulting in good search outcomes with less effort [52].

Similar conclusions are found in our experiment. Revealed in the qualitative themes, seekers demonstrate a positive attitude to serendipities. Seekers are surprised when they spot information that they did not anticipate on the screen and even show excitement if the information “hits” their needs. Although serendipity is often found in exploratory search [25,53], we argue that serendipity is relevant to both focused search and exploratory search because of the interleaving search approaches in health information seeking.

Serendipity associates with certain defining properties of health information seeking. First, general seekers are not experts in the health domain and do not have the knowledge to create proper search queries [6-8]. Observed in the study, they use the serendipitous findings as hints and directions for future searches. Also, serendipity is deemed as a way of making unforeseeable connections across different knowledge, and such connections are usually preferred, as people will favor using the least effort in finding information [54].

Curiosity is another factor to drive health information seeking, especially in the event of casual reading, for example, reading health information for self-learning rather than facing an immediate threat [17]. In this research, serendipity is found to trigger curiosity, and therefore, further readings are carried out. In many cases, casual seekers do not have a specific search target; hence, a fuzzy query approach (such as the design of BHX) can increase the chance of returning information that is interested to the user. This has been deemed as another manner approaching the information retrieval problem [55].

BHX gives an example of designing for serendipity. Instead of returning accurate search results as seen in most search engines, our design of providing a fuzzy search assists in health information exploration and enhances the user experience. With the advantages identified in this evaluation, we recommend to add serendipitous elements for health information seeking.

#### Considering Trust Implications

Trust is always an important component of health websites. Various guidelines have been proposed to ensure genuine and trustworthy online Web-based information [56,57]. Although these guidelines focus mainly on the quality and the accuracy of the content, the trust issues that arise from the information presentation and the UI are seldom discussed. In our study, we have identified new challenges for trustworthiness that result from the novel design.

By using a fuzzy approach for exploring information, it is inevitable that users will encounter some results that appear “weird” or “irrelevant.” From our observations, we identify 2 causes underlying this issue: the seeker does not recognize the connection between the displayed information and the current context or the user does not fully understand the meaning of the information visualization of the UI.

We have identified 2 extreme responses when seekers observe the questionable results. Some users trust the website and believe that they do not possess the expertise to understand why these results appear. They realize that the “irrelevant” result is an opportunity to learn the unknown and leads to serendipitous findings. For this scenario, as seekers may not be able to judge the validity of the results, research should focus on the accuracy of the algorithm for providing truly relevant information, even for serendipities.

Other seekers assume that the display of doubtful information is a fault of the system. These seekers often possess higher health literacy and more knowledge about the health issue, so they believe that the system gives incorrect information. Eventually the trust between seekers and the system will cease. For this type of seeker, the solution is to increase the transparency of how the system works, by clearly explaining the reasons behind the display of certain items in the UI. For example, the design can adopt different color codes or legends to indicate the results suggested by different heuristics or at different levels of confidence. Visual feedback (eg, highlights and animations) can also be added to represent the results originating from certain user activities.

Regardless of the circumstances, designers need to be careful in presenting health information when using new interaction techniques. User may have unexpected interpretations of the information presentation and the UI, as compared to what the designer expects. A comprehensive understanding of the users and usability tests can help to resolve such problems before release.

#### Enhancing Interactivity

This study shows that our design provides higher interactivity in comparison with traditional consumer health websites and therefore leads to higher user engagement and better user experience. This interactive nature gave users more options to retrieve more diverse information. Participants could manipulate this exploration through the UI, observe the changes to the results, and obtain new knowledge from this process. In contrast, the traditional experience relying on keyword search was seen as less playful and enjoyable. The positive findings are consistent with those of other research studies, which suggests that interactivity has a positive impact on information seeking [10,58,59]. This highlights the potentiality for enhancing the interactivity of health information websites.

In addition, interactivity and engagement may be related to better performance and higher rates of returning to a health website. According to Flow research, a person who enters a mental state of complete engagement and immersion into an activity will have higher success rate and will have a greater chance of reusing the system [60,61]. In the BHX study, participants were observed to engage with the elements supporting interactive exploration of health information. Therefore, we expect that a design with better interactivity will bring a positive outcome in seeking and learning consumer health information. Future research might focus on revealing the relationship among interactivity, engagement, and user revisits.

On the basis of our experience of this research, there are challenges to implement a website with these promising features. Introducing an interactive experience often requires a new UI. This may not be easily accepted by users and may have negative implications for trust as discussed previously. The existing content may not be directly usable in the new UI and would need to be manipulated to fit the distinct underlying model. For example, in our website, articles were processed with a computer program to generate the metadata for the sliders and had to be reviewed manually. Such additional work may be very time consuming and require significant effort. Hence, we should be aware of these challenges despite the better outcomes achieved by the proposed design.

### Limitations

Although most of our statistical tests are significant, the sample size is relatively small as compared with that of other quantitative studies. To reduce the impact, we applied a mixed approach to analyze both qualitative and quantitative data. In addition, the composition of the participants is mainly university members, which may not represent the general population of health information seekers. Future research will focus on a larger cohort of participants with a more diverse background.

Second, we evaluated all participants as a single group of health information seekers and did not capture attributes such as occupations and education level. For future research, studies focusing on different user groups may discover additional findings distinct to these groups.

Finally, only 1 website (ie, BHC) was chosen as the baseline, which limits the comparison only to the particular design of that website and therefore affects the generality of the results. Future work may evaluate the design implications in the context of other health websites or health information sources.

### Conclusions

In this paper, we present and evaluate BHX, which is a novel interface design that aims to address several problems faced by users in health information seeking. Through an observational study, we discover that the design of BHX can improve findability and discovery of information, as well as enhance the overall user experience. Moreover, the study shows that a mix of health information–seeking behaviors needs to be handled by health websites, highlighting the importance of providing specific support for these behaviors.

The positive results of this study reflect the importance of understanding different health information–seeking behaviors, as well as designing to accommodate these behaviors. Although previous eHealth research has suggested designing for users and their needs [5,22,23], this research takes a further step and proposes designing for behaviors, for example, reading, researching, and exploring. In this regard, 4 design considerations are emerged from our research. These considerations will lead to better support of the heterogeneous and shifting behaviors of health information seekers and ease the process of obtaining Web-based health information for users. Therefore, these elements should be applied in future designs and in HCI research.

Although promising outcomes are observed in this study, our future work includes addressing the critical feedback about the information presentation, extending the information sources to include other health databases, and investigating the effects of particular design features (eg, sliders and the exploration UI) in the health information–seeking processes.

## Appendix

Multimedia Appendix 1 Demonstration video of Better Health Explorer.

Multimedia Appendix 2 Task assignments of the participants.

Multimedia Appendix 3 Questions for guiding the semistructured interview.

Multimedia Appendix 4 Demographic and user interaction figures (N=31).

## Abbreviations

BHC: Better Health Channel
BHX: Better Health Explorer
HCI: Human-computer Interaction
UI: User interface
