# Supplementary material for: Designing Health Websites Based on Users’ Web-Based Information-Seeking Behaviors: A Mixed-Method Observational Study
Source: J Med Internet Res. 2016 Jun 6;18(6):e145. doi: 10.2196/jmir.5661 (PMC4914778; doi:10.2196/jmir.5661)
Supplement: Multimedia Appendix 3 [file jmir_v18i6e145_app3.pdf]

Interview Questions

1. How did these websites help you when you were uncertain what to look for?

2. How did you find new knowledge with these websites?

3. Do you think the diverse information presented to you was useful? Please explain.

4. Do you think the unexpected but interesting information presented to you was useful? Please explain.

5. Why did you feel engaged/not engaged with these websites?

6. Why did you feel enjoyable with the website?

7. Were there any differences in the way you approached these two tasks? Please explain.

8. How do you compare these two websites?

9. (For BHX Only) Do you think the design of this website (BHX), e.g. sliders, filters, animations…, was helpful or not helpful for the tasks? Why?
